# Irregular rupture propagation and geometric fault complexities during the 2010 Mw 7.2 El Mayor-Cucapah earthquake

**DOI:** 10.1038/s41598-022-08671-6

**Published:** 2022-03-17

**Authors:** Shinji Yamashita, Yuji Yagi, Ryo Okuwaki

**Affiliations:** 1 Graduate School of Science and Technology, University of Tsukuba, Tsukuba, Ibaraki 305-8572 Japan; 2 Faculty of Life and Environmental Sciences, University of Tsukuba, Tsukuba, Ibaraki 305-8572 Japan; 3 Mountain Science Center, University of Tsukuba, Tsukuba, Ibaraki 305-8572 Japan; 4 COMET, School of Earth and Environment, University of Leeds, Leeds, LS2 9JT UK

**Keywords:** Solid Earth sciences, Seismology

## Abstract

The 2010 *M*_W_ 7.2 El Mayor-Cucapah, Mexico, earthquake ruptured multiple faults with different faulting mechanisms. Resolving the earthquake rupture process and its relation to the geometric fault complexities is critical to our understanding of the earthquake source physics, but doing so by conventional finite-fault inversion is challenging because modelling errors due to inappropriate assumptions about the fault geometry distort the solution and make robust interpretation difficult. Here, using a potency density tensor approach to finite-fault inversion, we inverted the observed teleseismic *P* waveforms of the 2010 El Mayor-Cucapah earthquake to simultaneously estimate the rupture process and the fault geometry. We found that the earthquake consisted of an initial normal faulting rupture, which was followed by a strike-slip bilateral rupture towards the southeast and northwest that originated on the northwest side of the epicentre. The southeastern rupture propagated back through the initial rupture area, but with strike-slip faulting. Although the northwestern rupture propagated across the left step in the Puerta fault-accommodation zone, the rupture was temporarily stalled by the associated change of the fault geometry. These results highlight the irregular rupture process, which involved a back-propagating rupture and fluctuating rupture propagation controlled the complexity of the fault system.

## Introduction

**Figure 1:**
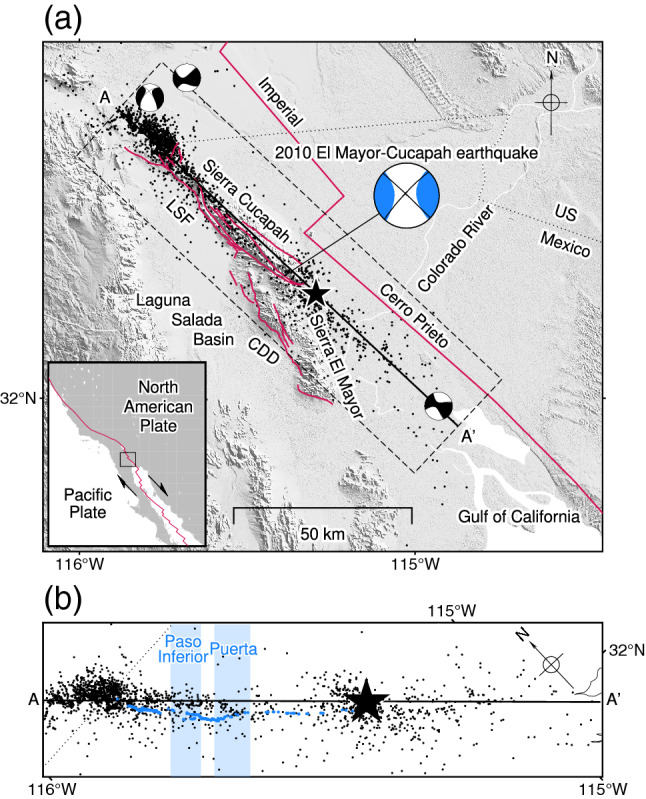
(**a**) Seismotectonic map of the study region. The blue and black beachballs indicate the GCMT solutions^10,11^ for the mainshock and 3-day aftershocks, respectively. The A–A′ line corresponds to the top of the model plane used for our optimum finite-fault solution. The star shows the epicentre of the 2010 El Mayor-Cucapah earthquake. Red lines indicate the plate-boundary fault^16^ and the Laguna Salada fault (LSF)–Cañada David detachment (CDD) fault system^17^. The inset shows the regional tectonic setting, and the dashed rectangle shows the area shown in (**b**). (**b**) Seismotectonic map of the area along A–A′. Blue dots represent surface faults^12^. Blue-shading highlights the Paso Inferior and Puerta accommodation zones^12^. The star is the relocated epicentre^13^. Black dots show relocated 3-day aftershocks^13^. The dotted line shows the U.S.–Mexico border. This figure was made with Generic Mapping Tools (v6.2.0)^18^ and the background topography in (**a**) is based on SRTMGL3 data^19^.

Geometric discontinuities in faults are known to cause irregular earthquake rupture behaviours, as evidenced by numerical fault models^1–5^ and observations of actual earthquakes^6–9^. Thus, earthquakes rupturing multiple faults provide a fruitful means of understanding the relationship between fault geometry and the rupture propagation process. One example is the 4 April 2010 *M*_W_ 7.2 El Mayor-Cucapah earthquake^10,11^, which ruptured at least seven faults^12^ in northeastern Baja California, Mexico, along the Pacific–North American plate margin (Fig. 1a). The epicentre was adjacent to the southeastern edge of the Sierra Cucapah (Fig. 1a)^13^. Major active fault systems near the epicentre include the Imperial and Cerro Prieto faults to the east and the Laguna Salada and Cañada David detachment faults to the west (Fig. 1a). However, these systems seem not to have ruptured during the 2010 El Mayor-Cucapah earthquake; rather, the earthquake occurred on multiple inactive or previously unrecognized faults^12,14^. The surface rupture trace of the 2010 earthquake, derived from interferometric synthetic aperture radar (InSAR) images and subpixel correlation, extends about 120 km towards the northwest and southeast, from near the U.S.–Mexico border in the north to the northern tip of the Gulf of California in the south, and shows right-lateral strike-slip displacement^14^. A field-based study documented detailed surface ruptures over ~ 55 km in length in the Sierra Cucapah, including the Paso Inferior and Puerta accommodation zones, which have formed between left steps of the main fault in the northern Sierra Cucapah (Fig. 1b)^12^. Relocated aftershocks distributed in the vicinity of the surface rupture and occurring within 3 days of the mainshock can be divided into three clusters: one near the U.S.–Mexico border, another around the epicentre^13^, and a third distributed between the epicentre and the northern edge of the Gulf of California (Fig. 1)^13,15^.

Several pioneering studies have constructed finite-fault models beneath the northwest–southeast-trending surface rupture by using InSAR images, satellite pixel offsets, and Global Navigation Satellite System data, but the number, arrangement, and orientation of the modelled faults vary among studies^14,20,21^; as a result, it is difficult to uniquely constrain the relationship between source process and fault geometry. Although the satellite geodetic data and field observations provide static information about the 2010 El Mayor-Cucapah earthquake, dynamic information such as the rupture propagation process must be estimated by a seismic waveform data analysis. The Global Centroid Moment Tensor (GCMT) solution^10,11^ of the mainshock shows a predominant strike-slip faulting mechanism, where the strike angle of the right-lateral nodal plane (313°) is consistent with the northwest–southeast orientation of the surface rupture (Fig. 1). In contrast, first-motion focal mechanisms estimated using *P*-waves predominantly show normal faulting^14,22^. This mechanism, which deviates from both the mainshock GCMT solution (Fig. 1a)^10,11^ and the right-lateral strike-slip surface rupture inferred from remote-sensing data^14^, suggests that (1) the faulting mechanism changed during the rupture^22^ and (2) the fault geometry at depth is complex^14^. Finite-fault inversion using waveform data alone^22^, as well as joint inversion using waveform data and geodetic data^14^, estimated a spatiotemporal slip-rate evolution on assumed fault planes comprising an almost north–south-striking and east-dipping fault with the epicentre, sandwiched between steeply dipping faults with northwest–southeast strikes to the north and south. Both inversion results show bilateral rupture on the northwest–southeast-striking faults about 20 s after the origin time^14,22^, but there are discrepancies in the detailed setting of the assumed fault geometry and the proposed rupture process. The inversion using strong-motion data shows a southeast propagating rupture before the bilateral rupture and repeated rupturing 5–40 km northwest of the epicentre after passage of the bilateral rupture^22^, whereas the joint inversion using teleseismic waveform and static data shows an initial rupture centred on the epicentre before the bilateral rupture and no repeated rupturing^14^. Because the fault geometry is prescribed and the slip-rate vector confined to the assumed model plane in finite-fault inversions, the inversion solution is heavily dependent on the predetermined strike and dip angles of the fault plane. Thus, an inconsistency between the true and assumed fault geometries is a major source of modelling errors, which bias the inversion solution^23,24^ and can lead to incorrect estimation of the rupture propagation direction^24^. Hence, it is challenging to apply the finite-fault inversion method to earthquakes for which the fault geometry is complex and not fully known.

One problem with the conventional finite-fault inversion approach is that the fault geometry prescribed by the modellers often does not represent the true fault geometry^23,24^. The resulting uncertainty of the fault geometry increases modelling errors^23,24^. Given that seismic waveform data has information on the fault geometry^24,25^, it would be desirable to let the data determine the fault geometry instead of prescribing the fault geometry a priori. Recently, a potency density tensor approach to finite-fault inversion that uses teleseismic *P* waveforms^24^ has been developed. In this approach, the fault orientation information inherent in the seismic waveform data is directly extracted as focal-mechanism variation projected onto an assumed model plane by increasing the degrees of freedom of the modelling, thereby mitigating modelling errors due to the uncertainty on the fault geometry^9,24,26–32^. Because this finite-fault inversion approach allows any type of fault slip, the estimated slip direction is independent of the predetermined orientation of the assumed model plane. Although teleseismic *P* waveforms are sensitive to the inferred fault orientation, they are insensitive to errors in the assumed source location^24^, which no longer requires a detailed assumption of the fault geometry. This finite-fault inversion approach explicitly introduces an error term of the Green’s function into the data covariance matrix^33^, which allows proper evaluation of the information in the observed data and prevents overfitting^33,34^. Thus, this inversion strategy is a data-driven strategy that enables the slip evolution and fault geometry to be estimated simultaneously without imposing a prescribed fault geometry^24^ or a non-negative slip constraint^24,33^, as is commonly used in conventional finite-fault inversions^35,36^. Thus, this finite-fault inversion approach is suitable for analysing the 2010 El Mayor-Cucapah earthquake, which ruptured multiple faults with difficult-to-prescribe geometries. Hereafter, we refer to this developed finite-fault inversion method as potency density tensor inversion because the slip amounts estimated with the focal mechanisms are represented by potency density tensors^37^ on the assumed model plane. Potency density tensor inversion stabilizes the solution by applying a spatiotemporal smoothing constraint^24^. The assumption of a global smoothing strength leads to excessive smoothing of parts where the moment releases change rapidly^27,28,31,32^, which may prevent proper estimation of the moment-rate function and focal-mechanism variation^32^. To avoid this problem, we applied a time-adaptive smoothing constraint that dynamically controls the smoothing strength to be inversely proportional to the amplitude of the potency-rate function, which contains temporal information about the slip-rate and the focal mechanism^32^. Hyper-parameters that control the smoothing strengths are objectively determined from observed data by using Akaike’s Bayesian Information Criterion (ABIC)^38,39^.

Adopting the time-adaptive smoothing constraint, we applied potency density tensor inversion to teleseismic *P* waveforms of the 2010 El Mayor-Cucapah earthquake and simultaneously estimated its spatiotemporal rupture evolution and focal-mechanism variation. The obtained source model shows an initial rupture with normal faulting around the epicentre followed by bilateral rupture with strike-slip faulting, which included irregular rupture behaviour caused by a discontinuity of the fault geometry. The results highlight fluctuations in rupture propagation controlled by the multiplicity and complexity of the fault system recorded in the observed teleseismic *P* waveforms.

## Data and model parameterization

**Figure 2:**
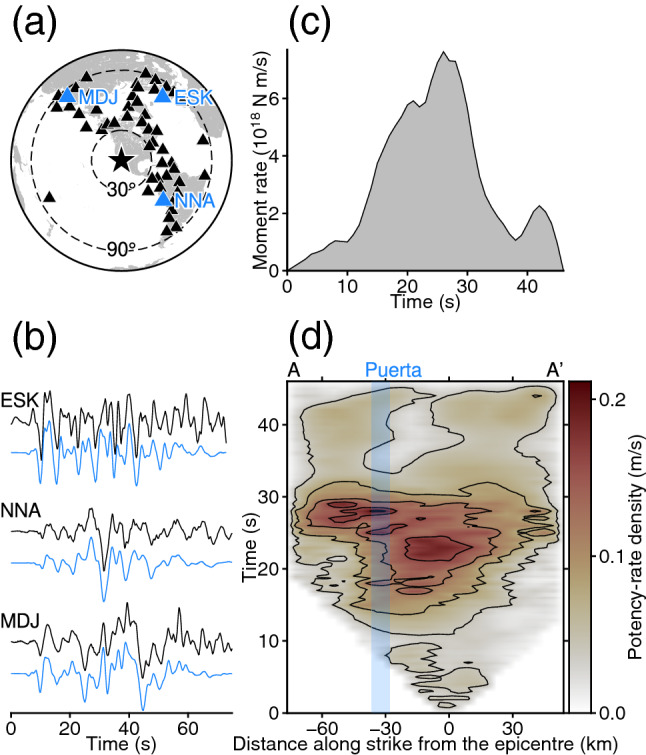
Summary of the inversion results. (**a**) Station distribution (triangles). Dashed circles show epicentral distances of 30° and 90°. Blue triangles indicate the stations used for the waveform fitting shown in (**b**). (**b**) Waveform fitting at the selected stations. The black and blue traces show the observed and synthesized waveforms, respectively, at each station (labelled with the station code). The sampling interval for plotting the waveforms is 0.05 s. (**c**) Moment-rate function. (**d**) Potency-rate density evolution (contour interval 0.04 m/s) projected along A–A′; distance is relative to the epicentre (Fig. 1a). The vertical blue-shaded bar shows the Puerta accommodation zone^12^. This figure was made with Generic Mapping Tools (v6.2.0)^18^.

We downloaded vertical-component *P* waveforms of the 2010 El Mayor-Cucapah earthquake globally observed at 52 stations at epicentral distances of 30°–90° via the Incorporated Research Institutions for Seismology Data Management Center (IRIS-DMC) (Fig. 2a). These 52 stations were selected due to their high signal-to-noise ratio^40^. As we will show in the “Results” section, the station coverage is adequate to resolve the rupture process in our model space, which is elongated along the northwest-southeast direction. We manually picked the *P*-wave first arrival and converted it into a velocity waveform to remove the seismograph response. The waveform data were then resampled at 1.0 s intervals. For the source region, we assumed a 1-D velocity structure derived from the CRUST1.0 model (see Supplementary Table S1)^41^ and calculated the theoretical Green’s functions by the method of Kikuchi and Kanamori^42^ at a sampling rate of 0.1 s. The attenuation time constraint *t*^∗^ for the *P*-wave was taken to be 1.0 s. We also performed an inversion near the epicentre using the 1-D velocity structure derived from the CRUST2.0 model (see Supplementary Table S2)^43^ and confirmed that our proposed source process was robust to the assumed velocity structure (see “Sensitivity and reproducibility tests”). In general, the application of a low-pass filter increases the off-diagonal components of the data covariance matrix^33^, which causes instability in obtaining the inverse of the data covariance matrix. Therefore, to stably find the inverse of the data covariance matrix, we did not apply a low-pass filter to either the observed waveforms or the Green’s functions^24,26–28,31,32^. As a by-product of this approach, we could estimate a seismic source model that could explain the observed waveforms undistorted by application of a low-pass filter (Fig. 2b, Supplementary Fig. S1).

**Figure 3:**
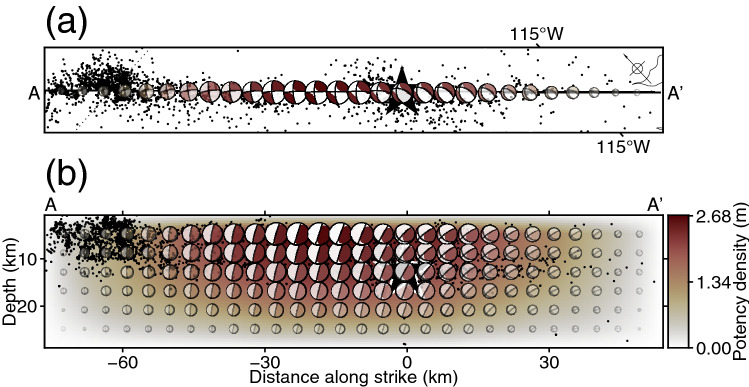
Potency density tensor distribution. (**a**) Potency density tensors at the top of the assumed model plane with a dip of 90°, projected onto an oblique map of the area along A–A′. The dotted line shows the U.S.–Mexico border. (**b**) Cross section of the potency density tensor distribution on the assumed model plane. The beachballs in (**b**) are shown in cross-sectional view from the southwest side. The star shows the hypocentre used for the inversion. Black dots show relocated seismicity 3 days after the mainshock^13^. This figure was made with Generic Mapping Tools (v6.2.0)^18^.

Potency density tensor inversion resolves a variable fault geometry on the assumed model plane to avoid arbitrariness in setting the model geometry. Although the spatial resolution of the teleseismic *P* waveform data is low, they contain information on the fault geometry^24,25^. Therefore, the method can stably estimate the variation of the focal mechanism contained in the data even on a flat model fault^24^. We adopted a model plane 130.5 km long × 28.0 km wide with a strike of 133° and a dip of 90° (Figs. 1, 3) based on the aftershock distribution^13,15^, the surface rupture traces^12,14,21,44^, and the mainshock GCMT solution^10,11^. We confirmed that the effect on the inversion solution of the selection of the model domain geometry was insignificant by conducting sensitivity tests with alternative settings, in which the model plane dipped 70° to the southwest or northeast (see “Sensitivity and reproducibility tests”). The initial rupture point was set at 32.264° N, 115.295° W and 12.8 km depth (Figs. 1, 3) by referring to the mainshock epicentre in the relocated earthquake catalog^13^ and the depth of the mainshock GCMT solution^10,11^. We expanded the potency-rate density function along the model plane into bilinear B-splines at spatial intervals of 4.5 km × 4.0 km in length and width and a temporal interval of 1.0 s. The maximum rupture-front velocity, which defines the starting time of rupture at each knot, was set to 3.65 km/s based on the *S*-wave velocity at the depth of the initial rupture point (see Supplementary Table S1)^41^. As we will show in the “Results” section, the obtained major potency-rate density is distributed behind the rupture-front, suggesting that this setting should be reasonable to discuss the multiplex rupture behaviour. The maximum source duration at each knot was set to 45 s based on previous inversion results for the 2010 earthquake^14,22^. Because the field survey reported that the rupture reached the ground surface^12^, we did not constrain the potency-rate density to zero at the top of the model plane. To assure modelling stability, which can be hampered by extreme discrepancies of the smoothing strengths in the finite-fault model, the lower bound of the referenced potency-rate function for the time-adaptive smoothing constraint was set to 20% of its maximum amplitude, following Yamashita et al.^32^. Potency density tensor inversion requires 2.5 times more model parameters than conventional finite-fault inversion because of the increased number of basis slip components^24^. Nonetheless, potency density tensor inversion prevents overfitting the observed data because it introduces a data covariance matrix to mitigate the effect of modelling errors originating from the uncertainty of the Green’s function^33,34^ and because it uses an ABIC-based approach to objectively determine the optimal relative weights of information from observed data and the smoothing constraint^33,38,39,45,46^.

## Results

The obtained potency density distribution for the 2010 El Mayor-Cucapah earthquake shows a major slip area distributed at depths shallower than 15 km and centred about 18 km northwest of the epicentre (Fig. 3). The spatial distribution of the focal mechanism shows that strike-slip faulting was dominant in the region northwest of the epicentre, whereas a dip-slip component became dominant towards the region southeast of the epicentre (Fig. 3). The moment-rate function shows that the major moment release started 10 s after the origin time, and two major peaks at 21 s and 26 s were followed by an independent minor peak at around 42 s (Fig. 2c). The obtained seismic moment was 1.30 × 10^20^ N m (*M*_W_ 7.3). The observed waveforms are well explained by the synthesized waveforms (Fig. 2b, Supplementary Fig. S1).

**Figure 4:**
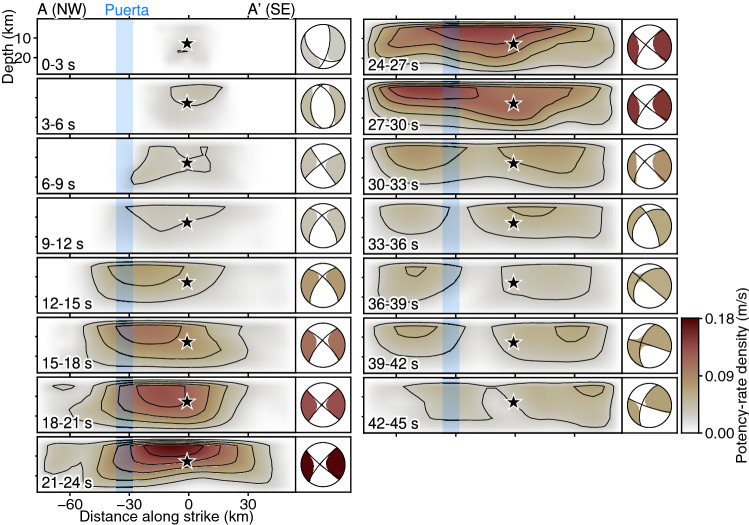
Snapshots of potency-rate density evolution. In each panel, a cross section of the potency-rate density distribution (contour interval 0.03 m/s) is shown on the left. The vertical blue-shaded bar shows the Puerta accommodation zone^12^. The star denotes the hypocentre used for the inversion. In each panel, the total moment tensor obtained by spatially integrating the potency-rate density tensors in the given time window is shown on the right. The colour corresponds to the maximum potency-rate density within the corresponding time window. The total moment tensors are shown as lower hemisphere projections onto the map with north upward. This figure was made with Generic Mapping Tools (v6.2.0)^18^.

**Figure 5:**
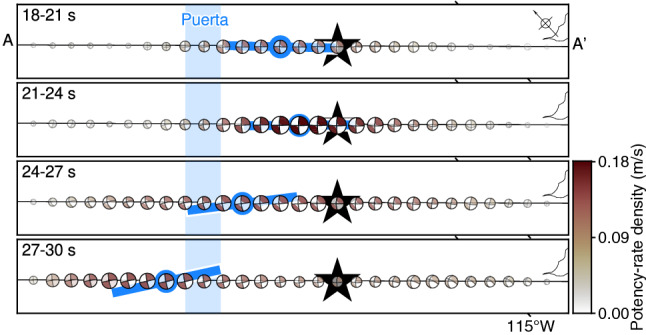
Selected snapshot of potency-rate density tensors at the top of the model plane, projected onto an oblique map of the area along A–A′. The blue line indicates the strike of the right-lateral nodal plane for the maximum potency-rate density. The vertical blue-shaded bar shows the Puerta accommodation zone^12^. The star denotes the epicentre used for the inversion. This figure was made with Generic Mapping Tools (v6.2.0)^18^.

The earthquake initiated around the epicentre and propagated mainly to the southeast until 6 s after the origin time (Figs. 2d, 4). The total moment tensor obtained by spatially integrating the potency-rate density tensors for the time window of each snapshot indicates that the initial rupture occurred with a normal faulting mechanism (Fig. 4). After 6 s, the rupture extended to about 20 km northwest of the epicentre, and the total moment tensor transitioned from normal to strike-slip faulting (Figs. 2d, 4). From 6 to 30 s, the strike-slip faulting rupture propagated bilaterally to the northwest and southeast along the right-lateral strike-slip nodal planes of the obtained focal mechanisms (Figs. 2d, 4, 5).

During the bilateral rupture episode, the southeastward rupture reached about 30 km southeast of the epicentre at 30 s (Figs. 2d, 4, 5). From 6 to 30 s, the southeastward rupture primarily showed a strike-slip faulting mechanism (Figs. 4, 5).

The northwestward rupture during the bilateral rupture episode reached about 30 km northwest of the epicentre by 12 s after the origin time and stagnated there until 21 s (Figs. 2d, 4). We note that the smaller amount of potency-rate density appears to keep propagating across 30 km northwest from the epicentre even after 12 s, which might be due to the application of smoothing to the potency-rate density. After temporarily stagnating at about 30 km northwest of the epicentre, the rupture evolved further northwestward until 30 s (Figs. 2d, 4). The potency-rate density tensors of the northwestward rupture exhibited a strike of 133° while the rupture was stagnating at 12–21 s on the right-lateral strike-slip nodal plane, and then the strike rotated about 10° anticlockwise during 21–30 s as the rupture advanced (Fig. 5).

After the main bilateral rupture, minor moment releases occurred in the northwestern and southeastern parts of the model domain (Fig. 2c,d). The obtained potency-rate density tensors show different focal mechanisms from those for the preceding bilateral rupture (Fig. 4). The rupture finally ceased 45 s after the origin time (Figs. 2d, 4).

## Sensitivity and reproducibility tests

**Figure 6:**
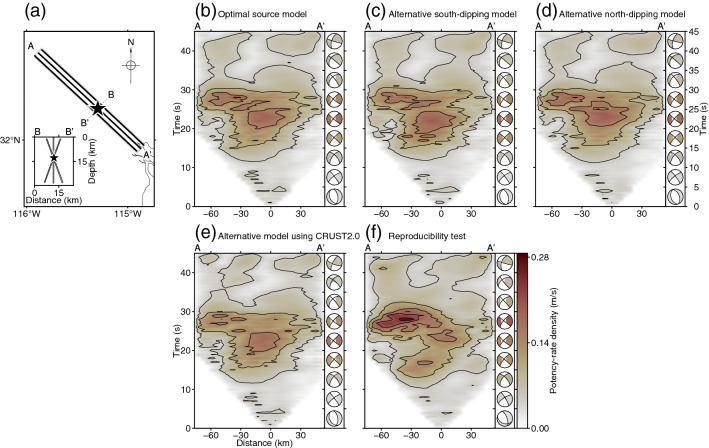
Summary of sensitivity and reproducibility test results. (**a**) Schematic map of the study area. The lines indicate the top of the model planes. The star is the epicentre used for the inversion. The inset shows a cross section along B–B′, and the lines show the model domains. In (**b**–**f**), potency-rate density evolution (contour interval 0.05 m/s) is projected along A–A′; distance is relative to the epicentre. (**b**) Our optimal solution obtained using a vertical model plane (i.e. the same as Fig. 2d). (**c**) The solution obtained using a plane dipping 70° southwest (strike 133°). (**d**) The solution obtained using a plane dipping 70° northeast (strike 313°). (**e**) The solution obtained using a vertical plane and the CRUST2.0 model^43^. (**f**) The solution obtained by inverting synthetic waveforms using our optimal solution as input. Beachballs to the right of each panel show the total moment tensors obtained in 5 s time windows. Note that the beachballs are shown as lower hemisphere projections onto the map with north upward. This figure was made with Generic Mapping Tools (v6.2.0)^18^.

To assess the robustness of the inversion solution to the assumed fault location and the assumption of a 1-D velocity structure near the epicentre, we inverted the observed teleseismic *P* waveforms after adopting alternative model settings. To test the sensitivity to fault location, we used two model planes, one dipping 70° to the southwest and the other dipping 70° to the northeast (Fig. 6a). To test the sensitivity to the assumed velocity structure, we inverted the observed waveforms in a vertical model plane using the CRUST2.0 structural velocity model (see Supplementary Table S2)^43^. For all sensitivity tests, we used the same model parameter settings as adopted in our preferred modelling, except for changing the model plane dip or structural velocity model.

We verified the reproducibility of the potency density tensor inversion by conducting numerical tests with our preferred solution as input. We generated synthetic waveforms for the 52 stations used in the inversion analysis by convolving our preferred inversion solution and the theoretical Green’s function with errors and background noise. Then, we inverted the synthetic waveforms using the same model parameter settings as adopted in our preferred modelling.

In both the sensitivity and reproducibility tests, all results reproduced the initial normal faulting rupture near the hypocentre during 0–5 s and the bilateral strike-slip faulting rupture during 5–30 s, including the rupture stagnation at about 30 km northwest of the epicentre, the anticlockwise rotation of the strike, and the subsequent advancement of the rupture at 20 s (Fig. 6, Supplementary Figs. S2, S3). The robustness of the sensitivity test results indicates that slight changes in the source location or the assumed near-source velocity structure do not affect the inversion solution of the teleseismic *P* waveforms. The consistency between the input and output models demonstrates the reproducibility of the method and supports the reliability of our proposed seismic source model.

In the alternative solution using the CRUST2.0 model^43^, a minor difference from the preferred solution is found during 35–45 s (Fig. 6b,e) that may undermine the certainty of our preferred solution during the very late rupture stage. In the reproducibility test result, the obtained potency-rate density tensors of the output obtained after about 30 s deviate from those of the input model, although the anticlockwise rotation of the strike at about 30 km northwest of the epicentre after 20 s remains robustly resolved (Fig. 6b,f, Supplementary Fig. S2). In general, modelling errors due to the uncertainty of the Green’s function increase with time^33^, which may contribute to these inconsistencies in the solutions at later rupture stages. In the following “Discussion”, therefore, we focus on the rupture propagation process only up to 30 s after the origin time, which seems to be reliably resolved based on the sensitivity and reproducibility tests.

## Discussion

Our source model derived from the teleseismic *P* waveforms shows that the 2010 El Mayor-Cucapah earthquake was initiated by a rupture on a normal fault (Fig. 4). Following the normal faulting rupture, a bilateral rupture originating about 20 km northwest of the epicentre began to propagate to the northwest and southeast, directionally coinciding with the strike of a right-lateral strike-slip nodal plane of the obtained focal mechanisms (Figs. 2d, 4, 5). The transition from normal faulting near the epicentre to strike-slip faulting starting northwest of the epicentre was clearly reproduced by both the sensitivity and reproducibility tests (see above section; Fig. 6); therefore, it is not an artifact of the inversion procedure or caused by modelling errors. The initial normal faulting rupture process is consistent with the first-motion focal mechanism derived by independent studies using *P*-waves^14,22^. The transition from the initial normal faulting and the subsequent bilateral rupture with strike-slip faulting obtained in our finite-fault solution explains the apparent discrepancy between the reported first-motion-based normal focal mechanism^14,22^ and the mainshock GCMT solution^10,11^ showing the strike-slip faulting mechanism.

During the strike-slip bilateral rupture episode, the southeastward propagating rupture started on the northwest side of the epicentre and had reached about 30 km southeast of the epicentre by 30 s after the origin time (Figs. 2d, 4). The faulting mechanism of the southeastward rupture was primarily strike-slip, even where it crossed the epicentre, and was distinctly different from the normal faulting mechanism of the initial rupture episode (Figs. 4, 5). The normal faulting mechanism appears around 30 s at 20–30 km southeast of the epicentre (Fig. 5), which might have ruptured the different fault segment than for the earlier strike-slip rupture. However, we note that the corresponding rupture signal is relatively weaker than that of the main strike-slip rupture episode, and the later phases from the earlier rupture can overlap, which may affect the solution at the later time. Both the mechanism and direction of this rupture were robustly resolved in the sensitivity and reproducibility tests (Fig. 6, Supplementary Figs. S2, S3). Our solution seems to show backward rupture propagation through the epicentre during the southeast rupture episode, but, given the different faulting mechanisms of the initial and southeast rupture episodes, it is likely that a different fault domain was ruptured during the latter than during the former; thus, the southeast rupture was not necessarily a re-rupture of the same fault. Instead of fixing the fault geometry a priori, we adopt the single model plane and directly solve the focal mechanism variation as data requires. As a result, we could find the multiplex rupture episodes among the different fault segments. However, it is still challenging for our current approach to rigorously differentiate whether the multiple ruptures, occurring at the similar location in a separate time, are on the same fault or multiple faults, if there are little focal mechanism variations among them. A similar back-propagating rupture towards the south from the north of the epicentre was also reported by a back-projection analysis using regional array data^47^. Another back-projection image using *S*-waves recorded by a local array also shows a high-frequency wave source moving from northwest to southeast of the epicentre between 20 and 30 s after the origin time^48^.

The northwestward propagating rupture during the strike-slip bilateral rupture episode propagated within a shallow domain (up to ~ 15 km deep) from 6 to 30 s after the origin time. Notably, the northwest rupture temporarily stagnated about 30 km northwest of the epicentre during 12–21 s before advancing farther northwestward during 21–30 s (Figs. 2d, 4). During this fluctuation of the rupture evolution, the potency-rate density tensors show right-lateral strike-slip faulting along the assumed model plane with a strike of 133° during the rupture stagnation before 21 s; then at 21–30 s, the strike rotates about 10° anticlockwise (Fig. 5). Both the sensitivity and reproducibility tests reproduced this rotation of the strike-slip faulting mechanism during the northwestward rupture (Supplementary Figs. S2, S3). The rupture stagnated at the eastern edge of the Puerta accommodation zone, where the faulting network is complex and includes a left-step in the surface rupture from south to north (Figs. 1b, 2d, 4, 5)^12^. Thus, the stagnation of the northwestward rupture occurred in a restraining bend caused by anticlockwise rotation of the strike of the right-lateral strike-slip fault on the northwest side of the epicentre. Theoretical studies have shown that ruptures can decelerate as they approach restraining bends^2,3,5^. Recent observational studies of the 2018 Palu, Indonesia, earthquake also detected acceleration of the rupture velocity beyond a fault bend^9,49^. Although we cannot determine the absolute fault location or rupture propagation velocity from the evolution of the potency-rate density tensor alone, the fault bend resolved in our solution may have controlled the stagnation and acceleration of the northwestward propagating rupture.

## Conclusions

We inverted teleseismic *P* waveforms of the 2010 El Mayor-Cucapah earthquake to simultaneously estimate the spatiotemporal evolution of the rupture and the focal mechanism by using the potency density tensor approach to finite-fault inversion. In the obtained source model, the rupture initiated with normal faulting, which was followed by a bilateral rupture with right-lateral strike-slip faulting that originated on the northwest side of the epicentre and propagated towards both the northwest and southeast. The southeastward bilateral rupture showed apparent back-rupture propagation, but, compared with the initial rupture episode, it ruptured a different fault domain and its faulting type differed. We also found that the northwestward bilateral rupture stagnated at around 30 km northwest of the epicentre because of a change in the strike of the associated fault geometry that coincides with a geometric discontinuity in the Puerta fault-accommodation zone. The potency density tensor approach to finite-fault inversion enabled us to conduct robust source modelling by suppressing modelling errors due to uncertainties on the fault geometry and to resolve the irregular rupture propagation controlled by the multiplicity and complexity of the fault system, which we were able to derive from the information included in the teleseismic *P* waveforms.

## Supplementary Information

Supplementary Information.

## Data Availability

All seismic data were downloaded through the IRIS Wilber 3 system (https://ds.iris.edu/wilber3/) or IRIS Web Services (https://service.iris.edu/), including the following seismic networks: (1) the Canadian National Seismograph Network (CN; https://doi.org/10.7914/SN/CN); (2) the Caribbean USGS Network (CU; https://doi.org/10.7914/SN/CU); (3) the GEOSCOPE (G; https://doi.org/10.18715/GEOSCOPE.G); (4) the GEOFON (GE; https://doi.org/10.14470/TR560404); (5) the Global Telemetered Seismograph Network (GT; https://doi.org/10.7914/SN/GT); (6) the New China Digital Seismograph Net- work (IC; https://doi.org/10.7914/SN/IC); (7) the IRIS/IDA Seismic Network (II; https://doi.org/10.7914/SN/II); (8) the Global Seismograph Network (IU; https://doi.org/10.7914/SN/IU). The moment tensor solutions are obtained from the GCMT catalog (https://www.globalcmt.org/CMTsearch.html). The relocated earthquake catalog is from the Southern California Earthquake Data Center website (https://scedc.caltech.edu/data/alt-2011-dd-hauksson-yang-shearer.html). The CRUST1.0 and CRUST2.0 structural velocity models are available through the websites https://igppweb.ucsd.edu/~gabi/crust1.html and https://igppweb.ucsd.edu/~gabi/crust2.html, respectively.

## References

[1] Harris RA, Day SM (1993). Dynamics of fault interaction: Parallel strike-slip faults. J. Geophys. Res. Solid Earth.

[2] Kase, Y. & Day, S. M. Spontaneous rupture processes on a bending fault. *Geophys. Res. Lett.* **33**, (2006).

[3] Duan B (2005). Multicycle dynamics of nonplanar strike-slip faults. J. Geophys. Res..

[4] Oglesby DD (2005). The dynamics of strike-slip step-overs with linking dip-slip faults. Bull. Seismol. Soc. Am..

[5] Bruhat L, Fang Z, Dunham EM (2016). Rupture complexity and the supershear transition on rough faults. J. Geophys. Res. Solid Earth.

[6] King G, Nábělek J (1985). Role of fault bends in the initiation and termination of earthquake rupture. Science (80-)..

[7] Okuwaki R, Yagi Y (2018). Role of geometric barriers in irregular-rupture evolution during the 2008 Wenchuan earthquake. Geophys. J. Int..

[8] Kehoe HL, Kiser ED (2020). Evidence of a supershear transition across a fault stepover. Geophys. Res. Lett..

[9] Okuwaki R, Hirano S, Yagi Y, Shimizu K (2020). Inchworm-like source evolution through a geometrically complex fault fueled persistent supershear rupture during the 2018 Palu Indonesia earthquake. Earth Planet. Sci. Lett..

[10] Dziewonski AM, Chou T-A, Woodhouse JH (1981). Determination of earthquake source parameters from waveform data for studies of global and regional seismicity. J. Geophys. Res. Solid Earth.

[11] Ekström G, Nettles M, Dziewoński AM (2012). The global CMT project 2004–2010: Centroid-moment tensors for 13,017 earthquakes. Phys. Earth Planet. Inter..

[12] Fletcher JM (2014). Assembly of a large earthquake from a complex fault system: Surface rupture kinematics of the 4 April 2010 El Mayor-Cucapah (Mexico) Mw 7.2 earthquake. Geosphere.

[13] Hauksson E, Yang W, Shearer PM (2012). Waveform relocated earthquake catalog for Southern California (1981 to June 2011). Bull. Seismol. Soc. Am..

[14] Wei S (2011). Superficial simplicity of the 2010 El Mayor-Cucapah earthquake of Baja California in Mexico. Nat. Geosci..

[15] Hauksson E (2011). The 2010 M w 7.2 El Mayor-Cucapah Earthquake Sequence, Baja California, Mexico and Southernmost California, USA: Active seismotectonics along the Mexican Pacific Margin. Pure Appl. Geophys..

[16] Bird, P. An updated digital model of plate boundaries. *Geochem. Geophys. Geosyst.* **4** (2003).

[17] SCEC Earthquake Response Content Management Site. Digitized Faults in the Sierral El Mayor and Sierra Cucapa. https://response.scec.org/node/273 (2010).

[18] Wessel P (2019). The Generic Mapping Tools Version 6. Geochem. Geophys. Geosyst..

[19] NASA JPL. NASA Shuttle Radar Topography Mission Global 3 arc second. NASA EOSDIS Land Process. DAAC 10.5067/MEaSUREs/SRTM/SRTMGL3.003 (2013).

[20] Xu X (2016). Refining the shallow slip deficit. Geophys. J. Int..

[21] Huang M (2017). Fault geometry inversion and slip distribution of the 2010 M w 7.2 El Mayor-Cucapah earthquake from geodetic data. J. Geophys. Res. Solid Earth.

[22] Uchide T, Yao H, Shearer PM (2013). Spatio-temporal distribution of fault slip and high-frequency radiation of the 2010 El Mayor-Cucapah, Mexico earthquake. J. Geophys. Res. Solid Earth.

[23] Ragon T, Sladen A, Simons M (2018). Accounting for uncertain fault geometry in earthquake source inversions—I: Theory and simplified application. Geophys. J. Int..

[24] Shimizu K, Yagi Y, Okuwaki R, Fukahata Y (2020). Development of an inversion method to extract information on fault geometry from teleseismic data. Geophys. J. Int..

[25] Shimizu K, Yagi Y, Okuwaki R, Fukahata Y (2020). Construction of fault geometry by finite-fault inversion of teleseismic data. Geophys. J. Int..

[26] Hicks SP (2020). Back-propagating supershear rupture in the 2016 Mw 7.1 Romanche transform fault earthquake. Nat. Geosci..

[27] Tadapansawut T, Okuwaki R, Yagi Y, Yamashita S (2021). Rupture process of the 2020 Caribbean Earthquake along the Oriente Transform Fault, involving supershear rupture and geometric complexity of fault. Geophys. Res. Lett..

[28] Yamashita S (2021). Consecutive ruptures on a complex conjugate fault system during the 2018 Gulf of Alaska earthquake. Sci. Rep..

[29] Hu Y, Yagi Y, Okuwaki R, Shimizu K (2021). Back-propagating rupture evolution within a curved slab during the 2019 Mw 8.0 Peru intraslab earthquake. Geophys. J. Int..

[30] Okuwaki R (2021). Illuminating a contorted slab with a complex intraslab rupture evolution during the 2021 Mw 7.3 East Cape, New Zealand earthquake. Geophys. Res. Lett..

[31] Tadapansawut, T., Yagi, Y., Okuwaki, R., Yamashita, S. & Shimizu, K. Conjugate and bending faults drive the multiplex ruptures during the 2014 Mw 6.2 Thailand earthquake. 10.31223/X56P7T.

[32] Yamashita, S., Yagi, Y., Okuwaki, R. & Shimizu, K. Potency density tensor inversion of complex body waveforms with time-adaptive smoothing constraint. 10.31223/X5JW4V.

[33] Yagi Y, Fukahata Y (2011). Introduction of uncertainty of Green’s function into waveform inversion for seismic source processes. Geophys. J. Int..

[34] Duputel Z, Agram PS, Simons M, Minson SE, Beck JL (2014). Accounting for prediction uncertainty when inferring subsurface fault slip. Geophys. J. Int..

[35] Hartzell SH, Heaton TH (1983). Inversion of strong ground motion and teleseismic waveform data for the fault rupture history of the 1979 Imperial Valley, California, earthquake. Bull. Seismol. Soc. Am..

[36] Das S, Kostrov BV (1990). Inversion for seismic slip rate history and distribution with stabilizing constraints: Application to the 1986 Andreanof Islands Earthquake. J. Geophys. Res..

[37] Ampuero J-P (2005). Ambiguity of the moment tensor. Bull. Seismol. Soc. Am..

[38] Akaike H (1980). Likelihood and the Bayes procedure. Trab. Estad. Y Investig. Oper..

[39] Yabuki T, Matsu’ura M (1992). Geodetic data inversion using a Bayesian information criterion for spatial distribution of fault slip. Geophys. J. Int..

[40] Okuwaki R, Yagi Y, Aránguiz R, González J, González G (2016). Rupture process during the 2015 Illapel, Chile Earthquake: Zigzag-Along-Dip rupture episodes. Pure Appl. Geophys..

[41] Laske G, Masters G, Ma Z, Pasyanos M (2013). Update on CRUST1.0—A 1-degree global model of Earth’s crust. EGU Gen. Assem..

[42] Kikuchi M, Kanamori H (1991). Inversion of complex body waves—III. Bull. Seismol. Soc. Am..

[43] Bassin C, Laske G, Masters G (2000). The current limits of resolution for surface wave tomography in North America. EOS Trans. AGU.

[44] Oskin ME (2012). Near-field deformation from the El Mayor-Cucapah earthquake revealed by differential LIDAR. Science (80-)..

[45] Fukahata Y, Yagi Y, Matsu’ura M (2003). Waveform inversion for seismic source processes using ABIC with two sorts of prior constraints: Comparison between proper and improper formulations. Geophys. Res. Lett..

[46] Fukahata Y, Nishitani A, Matsu’ura M (2004). Geodetic data inversion using ABIC to estimate slip history during one earthquake cycle with viscoelastic slip-response functions. Geophys. J. Int..

[47] Meng, L., Ampuero, J. P., Page, M. T. & Hudnut, K. W. Seismological evidence and dynamic model of reverse rupture propagation during the 2010 M7.2 El Mayor Cucapah earthquake. In *AGU Fall Meeting Abstracts* vol. 2011 S52B-04 (2011).

[48] Meng L, Allen RM, Ampuero JP (2014). Application of seismic array processing to Earthquake early warning. Bull. Seismol. Soc. Am..

[49] Bao H (2019). Early and persistent supershear rupture of the 2018 magnitude 7.5 Palu earthquake. Nat. Geosci..

[50] Gasperini P, Vannucci G (2003). FPSPACK: A package of FORTRAN subroutines to manage earthquake focal mechanism data. Comput. Geosci..

