# Supplementary material for: Irregular rupture propagation and geometric fault complexities during the 2010 Mw 7.2 El Mayor-Cucapah earthquake
Source: Sci Rep. 2022 Mar 17;12:4575. doi: 10.1038/s41598-022-08671-6 (PMC8931087; doi:10.1038/s41598-022-08671-6)
Supplement: Supplementary file 1 — Supplementary Information. [file 41598_2022_8671_MOESM1_ESM.pdf]

Supporting Information for

Irregular rupture propagation and geometric fault complexities during the 2010 Mw 7.2 El Mayor-Cucapah earthquake

Shinji Yamashita[1], Yuji Yagi[2], and Ryo Okuwaki[2,3,4]

[1]Graduate School of Science and Technology, University of Tsukuba, Tsukuba, Ibaraki 305-8572, Japan.

[2]Faculty of Life and Environmental Sciences, University of Tsukuba, Tsukuba, Ibaraki 305-8572, Japan.

[3]Mountain Science Center, University of Tsukuba, Tsukuba, Ibaraki 305-8572, Japan.

[4]COMET, School of Earth and Environment, University of Leeds LS2 9JT, UK.

Contents

Tables S1 and **S2**. Velocity structure models used for calculating the Green's function.

Figure S1. Waveform fitting between observed and synthesized waveforms.

Figure S2. Snapshots of potency-rate density evolution for reproducibility test.

Figure S3. Snapshots of potency-rate density evolution for sensitivity tests.

Table S1. CRUST1.0 structural velocity model[1] used to calculate the Green's function.

V_P (km/s)	V_S (km/s)	Density (10^3 kg/m^3)	Thickness (km)
6.10	3.55	2.74	9.28
6.30	3.65	2.78	7.96
6.60	3.60	2.86	8.20
8.05	4.47	3.32	0.00

Table S2. CRUST2.0 structural velocity model[2] used to calculate the Green's function.

V_P (km/s)	V_S (km/s)	Density (10^3 kg/m^3)	Thickness (km)
6.10	3.50	2.75	10.50
6.30	3.60	2.80	8.50
6.60	3.60	2.90	8.50
8.00	4.60	3.30	0.00

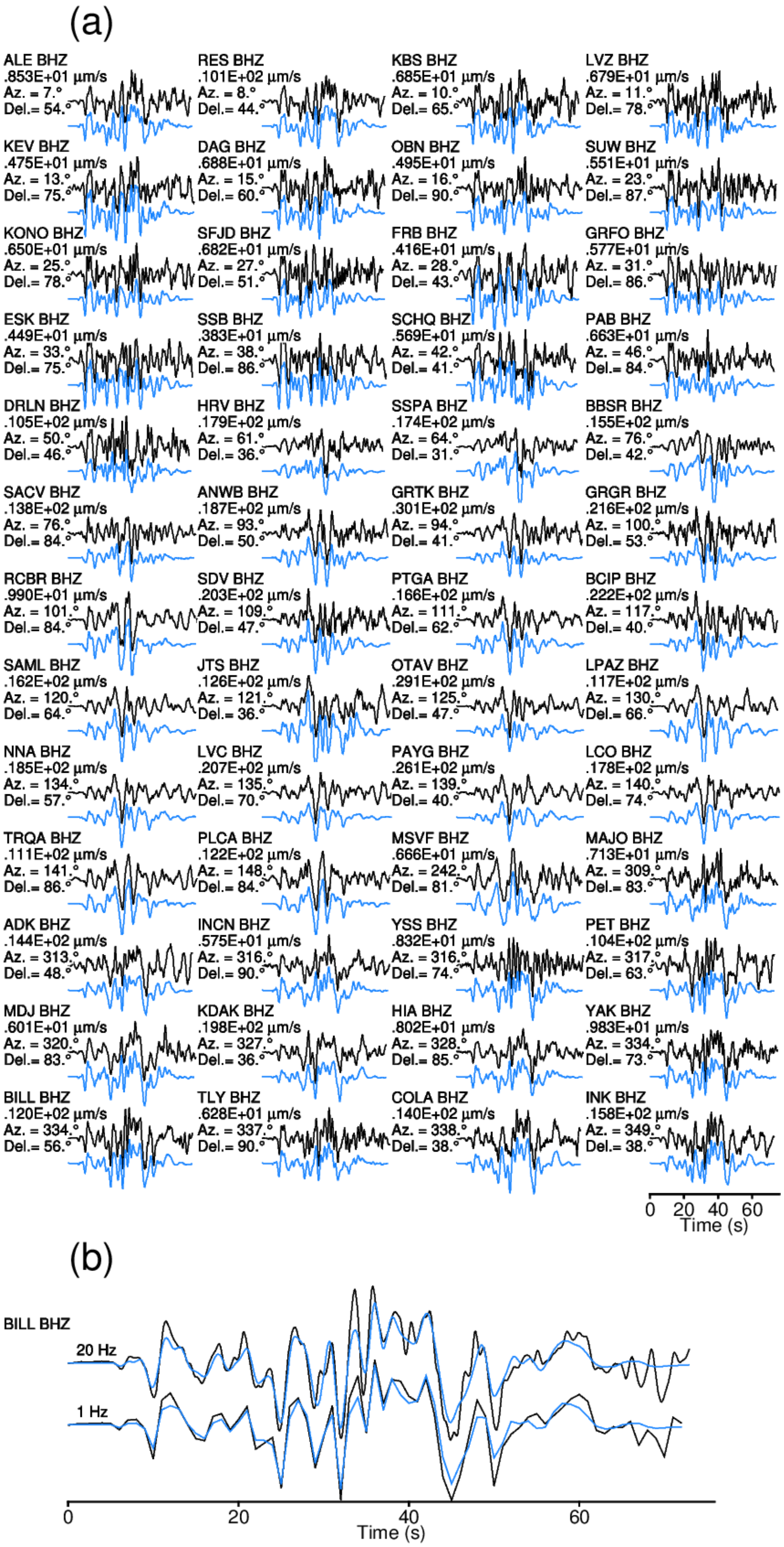
(a)
ALE BHZ
.853E+01 µm/s
Az. = 7.°
Del.= 54.°
RES BHZ
.101E+02 µm/s
Az. = 8.°
Del.= 44.°
KBS BHZ
.685E+01 µm/s
Az. = 10.°
Del.= 65.°
LVZ BHZ
.679E+01 µm/s
Az. = 11.°
Del.= 78.°
KEV BHZ
.475E+01 µm/s
Az. = 13.°
Del.= 75.°
DAG BHZ
.688E+01 µm/s
Az. = 15.°
Del.= 60.°
OBN BHZ
.495E+01 µm/s
Az. = 16.°
Del.= 90.°
SUW BHZ
.551E+01 µm/s
Az. = 23.°
Del.= 87.°
KONO BHZ
.650E+01 µm/s
Az. = 25.°
Del.= 78.°
SFJD BHZ
.682E+01 µm/s
Az. = 27.°
Del.= 51.°
FRB BHZ
.416E+01 µm/s
Az. = 28.°
Del.= 43.°
GRFO BHZ
.577E+01 µm/s
Az. = 31.°
Del.= 86.°
ESK BHZ
.449E+01 µm/s
Az. = 33.°
Del.= 75.°
SSB BHZ
.383E+01 µm/s
Az. = 38.°
Del.= 86.°
SCHQ BHZ
.569E+01 µm/s
Az. = 42.°
Del.= 41.°
PAB BHZ
.663E+01 µm/s
Az. = 46.°
Del.= 84.°
DRLN BHZ
.105E+02 µm/s
Az. = 50.°
Del.= 46.°
HRV BHZ
.179E+02 µm/s
Az. = 61.°
Del.= 36.°
SSPA BHZ
.174E+02 µm/s
Az. = 64.°
Del.= 31.°
BBSR BHZ
.155E+02 µm/s
Az. = 76.°
Del.= 42.°
SACV BHZ
.138E+02 µm/s
Az. = 76.°
Del.= 84.°
ANWB BHZ
.187E+02 µm/s
Az. = 93.°
Del.= 50.°
GRTK BHZ
.301E+02 µm/s
Az. = 94.°
Del.= 41.°
GRGR BHZ
.216E+02 µm/s
Az. = 100.°
Del.= 53.°
RCBR BHZ
.990E+01 µm/s
Az. = 101.°
Del.= 84.°
SDV BHZ
.203E+02 µm/s
Az. = 109.°
Del.= 47.°
PTGA BHZ
.166E+02 µm/s
Az. = 111.°
Del.= 62.°
BCIP BHZ
.222E+02 µm/s
Az. = 117.°
Del.= 40.°
SAML BHZ
.162E+02 µm/s
Az. = 120.°
Del.= 64.°
JTS BHZ
.126E+02 µm/s
Az. = 121.°
Del.= 36.°
OTAV BHZ
.291E+02 µm/s
Az. = 125.°
Del.= 47.°
LPAZ BHZ
.117E+02 µm/s
Az. = 130.°
Del.= 66.°
NNA BHZ
.185E+02 µm/s
Az. = 134.°
Del.= 57.°
LVC BHZ
.207E+02 µm/s
Az. = 135.°
Del.= 70.°
PAYG BHZ
.261E+02 µm/s
Az. = 139.°
Del.= 40.°
LCO BHZ
.178E+02 µm/s
Az. = 140.°
Del.= 74.°
TRQA BHZ
.111E+02 µm/s
Az. = 141.°
Del.= 86.°
PLCA BHZ
.122E+02 µm/s
Az. = 148.°
Del.= 84.°
MSVF BHZ
.666E+01 µm/s
Az. = 242.°
Del.= 81.°
MAJO BHZ
.713E+01 µm/s
Az. = 309.°
Del.= 83.°
ADK BHZ
.144E+02 µm/s
Az. = 313.°
Del.= 48.°
INCN BHZ
.575E+01 µm/s
Az. = 316.°
Del.= 90.°
YSS BHZ
.832E+01 µm/s
Az. = 316.°
Del.= 74.°
PET BHZ
.104E+02 µm/s
Az. = 317.°
Del.= 63.°
MDJ BHZ
.601E+01 µm/s
Az. = 320.°
Del.= 83.°
KDAK BHZ
.198E+02 µm/s
Az. = 327.°
Del.= 36.°
HIA BHZ
.802E+01 µm/s
Az. = 328.°
Del.= 85.°
YAK BHZ
.983E+01 µm/s
Az. = 334.°
Del.= 73.°
BILL BHZ
.120E+02 µm/s
Az. = 334.°
Del.= 56.°
TLY BHZ
.628E+01 µm/s
Az. = 337.°
Del.= 90.°
COLA BHZ
.140E+02 µm/s
Az. = 338.°
Del.= 38.°
INK BHZ
.158E+02 µm/s
Az. = 349.°
Del.= 38.°
0 20 40 60
Time (s)
(b)
BILL BHZ
20 Hz
1 Hz
0
20
40
60
Time (s)

Figure S1. Waveform fitting for our optimal solution. (a) Comparison of observed waveforms (upper black trace) with synthesized waveforms (lower blue trace) at all stations. Each panel is labeled with the station name, maximum amplitude, azimuth (Azi.), and epicentral distance (Del.) from the mainshock. The waveforms were resampled to 20 Hz for plotting. (b) Comparison of observed (black) and synthesized (blue) waveforms at BILL plotted at 20 Hz (upper) and 1 Hz (lower) sampling, which the latter is the calculation interval of the potency-rate density function of the inversion. This figure was made with Generic Mapping Tools (v6.2.0)[3].

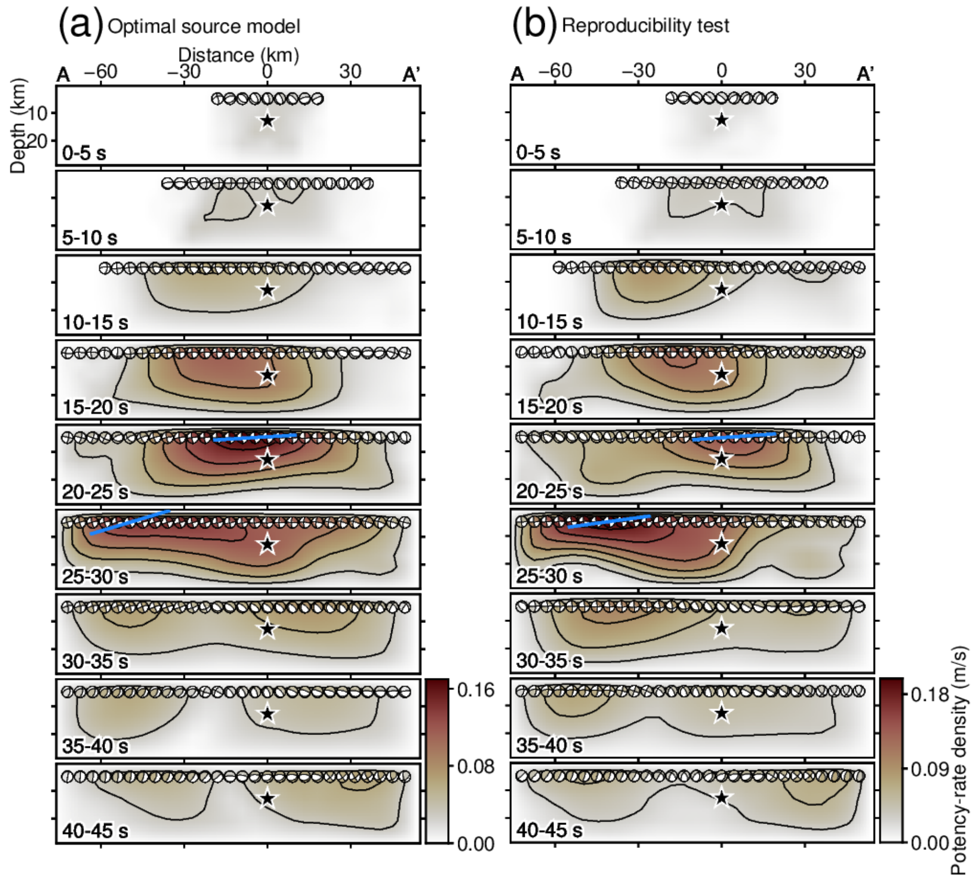

Figure S2. Snapshots of potency-rate density evolution for reproducibility test. (a) Our optimal solution obtained using a vertical model plane. (b) The solution obtained by inverting synthetic waveforms using our optimal solution as input. The legends are similar to Fig. 4. This figure was made with Generic Mapping Tools (v6.2.0)[3].

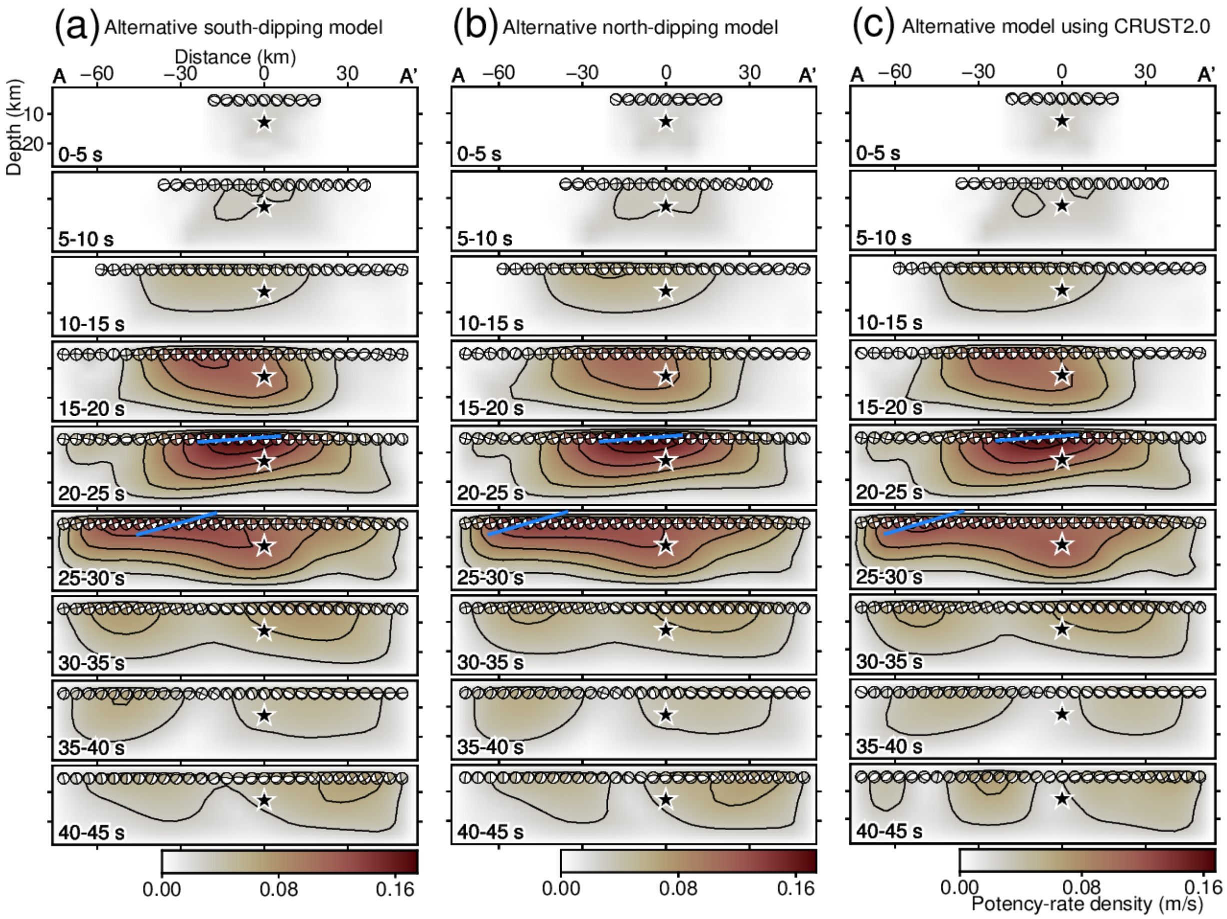

Figure S3. Snapshots of potency-rate density evolution for sensitivity tests. (a) The solution obtained using a plane dipping 70° southwest (strike 133°). (b) The solution obtained using a plane dipping 70° northeast (strike 313°). (c) The solution obtained using a vertical plane and the CRUST2.0 model (Table S2)[2]. The legends are similar to Fig. 4. This figure was made with Generic Mapping Tools (v6.2.0)[3].

References

1. Laske, G., Masters, G., Ma, Z. & Pasyanos, M. Update on CRUST1.0---A 1-degree global model of Earth's crust. *EGU Gen. Assem. 2013* **15**, 2658 (2013).

2. Bassin, C., Laske, G. & Masters, G. The current limits of resolution for surface wave tomography in North America. *EOS Trans AGU* **81**, F897 (2000).

3. Wessel, P. *et al.* The Generic Mapping Tools Version 6. *Geochemistry, Geophys. Geosystems* **20**, 5556–5564 (2019).
